# Morphological and Color Differences between Island and Mainland Populations in the Mexican Red Rump Tarantula, *Brachypelma vagans*

**DOI:** 10.1673/031.013.9501

**Published:** 2013-09-30

**Authors:** Claudia A. Vilchis-Nestor, Salima Machkour-M'Rabet, Irene de los A. Barriga-Sosa, Peter Winterton, Yann Hénaut

**Affiliations:** 1 Bioconservación ante el Cambio Global, El Colegio de la Frontera Sur (ECOSUR), Avenida Centenario Km 5.5, AP 424, 77014 Chetumal, Quintana Roo, Mexico; 2 Planta Experimental de Producción Acuícola, Departamento de Hidrobiología, División de Ciencias Biológicas y de la Salud, Universidad Autónoma Metropolitana-lztapalapa, Mexico; 3 Université de Toulouse - Paul Sabatier, 118, Route de Narbonne, F-31062 Toulouse cedex 4, France

**Keywords:** color polymorphism, introduced species, morphometric

## Abstract

The introduction of species into new ecosystems, especially in small and isolated regions such as islands, offers an excellent opportunity to answer questions of the evolutionary processes occurring in natural conditions on a scale that could never be achieved in laboratory conditions. In this study, we examined the Mexican red rump tarantula *Brachypelma vagans* Ausserer (Mygalomorphae: Theraphosidae), a species that was introduced to Cozumel Island, Mexico, 40 years ago. This introduction provides an exceptional model to study effects such as morphological variation between island populations and those on the mainland in open habitats facing the island. Intraspecific variation related to the color polymorphism was compared. The aim of this study was to determine the phenotypic differences between continental populations of *B. vagans* and the introduced population on Cozumel Island. Phenotypic difference was evaluated using two approaches: 1) comparison of the morphometric measurements of adult and juvenile individuals at the local scale and between continental and island populations, and 2) comparison of individual color polymorphism between mainland and island populations. Two locations were sampled within the continental part of the Yucatan peninsula and two on the island of Cozumel. The number of samples analyzed at each site was 30 individuals. The morphometric results showed significant differences between continental and island populations, with bigger individuals on the island. In addition, three new variations of the typical color pattern of *B. vagans* recorded so far were observed. This study opens the door to further investigations to elucidate the origin of the phenotypic variation of the isolated individuals on Cozumel Island. Also, the widest range of color morphs found for a tarantula species is reported.

## Introduction

The true tarantulas are distributed in tropical regions around the world and currently about 937 species have been identified (Platnick 2011). Mexico is ranked second worldwide in tarantula diversity with more than 64 species (Platnick 2011).

The genus *Brachypelma* is distributed in approximately 30% of the Mexican territory and is represented by 14 species (*B. annitha*, *B. albiceps*, *B. auratum*, *B. baumgarteni*, *B. boehmei*, *B. emilia*, *B. hamorii*, *B. khlenbergi*, *B. klaasi*, *B. schroederi*, *B. smithi*, *B. vagans*, *B. verdezi*), including one species endemic to the extreme north of the Yucatan peninsula (*B. epicureanum*) (Smith 1994; Platnick 2011).

The Mexican red rump tarantula, *Brachypelma vagans* Ausserer (Mygalomorphae: Theraphosidae), is a nocturnal predator, feeding mainly on ground-dwelling arthropods and small vertebrates (Marshall 1996). Although it is a solitary spider, like all other tarantulas, it lives in clustered colonies in areas modified by human activities, where it can occur in high densities (Machkour-M'Rabet et al. 2005). This fossorial tarantula builds a deep burrow (from 20 cm to 40 cm) mainly in clay soils and emerges at night in search of food (Machkour-M'Rabet et al. 2007). The burrow has a single entrance closed with silk during the day, which reduces water loss and allows the detection of vibrations from potential prey (Hamilton 2008). It is composed of one or more chambers that serve as nests, shelter, molting chamber, and larder (Machkour-M'Rabet et al. 2007). Growth is slow, with one molt per year, and sexual maturity is reached late, at around the 7th year). Every year, a mature female can produce 1 egg sac (or clutch) with a diameter of 5 cm and from which approximately 300 spiders emerge (Moore 1994). Juveniles remain with the mother for a few weeks before dispersing together in a typical single file structure (Reichling 2000; Dor et al. 2008). Unfortunately, the survival of the juveniles during their dispersal is limited because they suffer a high predation rate from other spiders (Dor and Hénaut 2011). Also, adults suffer cannibalism (Hénaut and Machkour-M'Rabet 2005), particularly among females (Dor et al. 2008). A recent genetic study (Machkour-M'Rabet et al. 2012) confirmed this species had a population structure with multiple and very well-defined local populations in which the females show high fidelity to their natal sites (Longhorn et al. 2007). Males, on the other hand, began their reproduction phase by walking over an extremely wide territory, guaranteeing the dispersion of the genetic pool. This behavior leads *B. vagans* to present a metapopulation structure on the mainland (Campeche and Quintana Roo States, Mexico). Furthermore, the genetic study of Machkour-M'Rabet et al. (2012) indicated that the introduction of a few *B. vagans* individuals to Cozumel Island in 1971 (Martínez-Morales and Cuarón 1999) led to the establishment of a new isolated population with a loss of genetic diversity due to the founder effect.

Generally, the introduction of species into new regions is considered to have a negative impact on native biodiversity and cause extensive economic and/or ecological damage (Vitousek et al. 1996; Clavero and García-Bertou 2005). These negative effects are more important in small and isolated regions such as island habitats (Gillespie and Roderick 2002).

Nevertheless, invasive species are considered to be an opportunity to understand a range of basic research questions, such as: 1) how fundamental ecological and evolutionary processes occur in real time, 2) determining the rate of processes (e.g., genetic changes) that are difficult to obtain in native species, 3) providing data that would often be deemed unethical to be collected in a planned experiment, and 4) providing unplanned experiments across large spatial and temporal scales that are often approximate replicas across islands and entire continents (Sax et al. 2007).

The recent introduction of species to islands (naturally or by any human process) can offer a unique opportunity to study the dynamics of diversification and the intraspecific variation of responses to insularity (Sax et al. 2007; Mathys and Lockwood 2011). The evolutionary responses to new habitats (e.g., island introduction) can occur rapidly (within a few years), as demonstrated under laboratory conditions (Rice and Hostert 1993) but also in natural settings without anthropogenic selective pressure (Stockwell et al. 2003; Cox 2004).

The introduction of *B. vagans* only 40 years ago on Cozumel Island (Quintana Roo, Mexico; Martínez-Morales and Cuarón 1999) offers a great opportunity to study different evolutionary mechanisms in real time. One of the evolutionary changes that can be studied is the morphological variation between island and mainland populations. Very few studies present information on morphological variation in tarantula species. Shillington and Peterson (2002) observed that males of *Aphonopelma anax* have a smaller abdomen and longer legs than females. Machkour-M'Rabet et al. (2005) performed a morphometric study among *B. vagans* females, males, and juveniles in a limited geographic area but did not report differences between males and females; however, the low number of adult individuals included in the study (females: n = 11; males: n = 6) may explain the low probability of detecting statistically significant differences. Our study was aimed at determining morphological variation in island and mainland populations of the tarantula *B. vagans*.

An interesting aspect of evolutionary change is intraspecific color morph variation. Color polymorphism and pattern variation has been widely reported for many spider species, and different reviews summarize what is known about the evolutionary and ecological significance of morph variation (Oxford and Gillespie 1998, 2001; Oxford 2005; Roulin and Bize 2007; Oxford 2009). Color polymorphism is a genetically determined trait that relates to a small number of major loci and provides an evident link between a genotype and the expressed phenotype (Oxford and Gillespie 2001). Color and pattern variation can be associated to different evolutionary mechanisms such as sexual selection, natural selection process, adaptation to different habitats, avoidance of predation, and thermoregulation, among others (Oxford and Gillespie 1998, 2001; Croucher et al. 2011). In most studies of spider color polymorphism, the number of morphs is typically 2 or 3; exceptionally, the Hawaiian happy-face spider, *Theridion grallator*, exhibited more than 20 described abdominal color patterns with a palette of yellow, red, white, and black pigments (Oxford and Gillespie 1996a, b, 2001). Recently, a new case of an exuberant number of color morphs was identified in *Theridion californicum*, with a least 11 distinct color patterns (Oxford 2009). Tarantula species can present some intraspecific variation in their coloration, but this has never been studied in detail. West (2005) reported two variations for color patterns of *B. vagans*: 1) body entirely velvet black with long orangey red hairs on the abdomen, and 2) a wide buff-colored fringe around the prosoma. But, our field observations suggested a large panel of possible color morphs for this tarantula species that must be described and analyzed to understand their origin.

**Figure 1.**
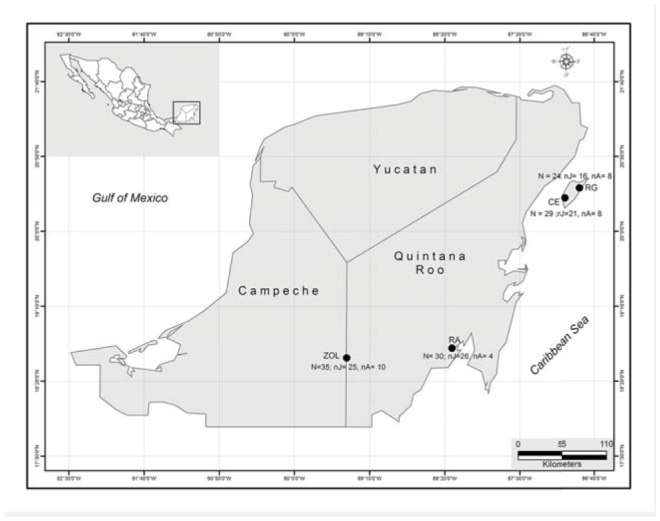
Collecting sites for *Brachypelma vagans*. N: total number of individuals collected; nA: number of adults; nj: number of juveniles; RA: Raudales; RG: Rancho Guadalupe; CE: El Cedral; ZOL: Zho-Laguna. High quality figures are available online.

Thus, the main goal of our study was to determine the phenotypic variation between continental populations of *B. vagans* and the recently founded populations on Cozumel Island in the Mexican Caribbean. Phenotypic variation was evaluated using two approaches: 1) comparison of morphometric measurements of adults and juveniles at the local population level and between continental and island populations, and 2) color polymorphism in each local population, with comparisons between continental and island populations.

## Materials and Methods

### Study Area

The study was conducted from February to March 2011 in four rural communities in the Yucatan peninsula (southern Mexico) (Figure 1). Three villages were located in the State of Quintana Roo: 1) Raudales (18° 42′ N, 88° 15′ W), a continental village, 2) Rancho Guadalupe, in the north of Cozumel Island (20° 29′ N, 86° 50′ W), and 3) El Cedral, in the south of Cozumel Island (20° 21′ N, 86° 39′ W). One village, Zoh-Laguna, was situated in Campeche State (18° 35′ N, 89° 24′ W) (Figure 1). Both continental communities (Raudales and Zoh-Laguna) were selected for their high density of tarantulas, as observed previously by Machkour-M'Rabet et al. (2005, 2007, 2009). The sites on Cozumel Island were selected because they are near the place where individuals of *B. vagans* were released 40 years ago after having been brought to the island by a film production company (Martinez-Morales and Cuarón 1999).

### Morphometric measurements

Around 30 individuals (juveniles and adults) were collected at each locality (see details in Figure 1). They were all found by talking with the village residents, who indicated locations of possible tarantulas. Each of the sites indicated (generally backyards, gardens, paths) was exhaustively searched to find the burrow entrances. All parts of the village were browsed.

**Figure 2.**
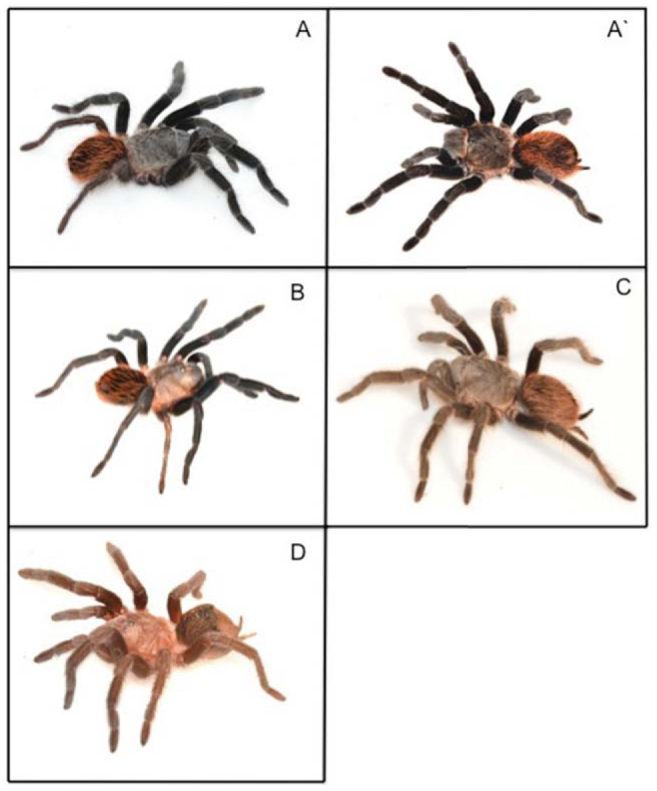
Color polymorphism found in the different populations of *Brachypelma vagans*. 2A and 2A': typical color patterns described by West (2005); 2B: abdomen orange; 2C: black-brown color; 2D: pink-red color (for detailed description of these color patterns see Materials and Methods). High quality figures are available online.

For each individual, the following data was recorded, as described in Machkour-M'Rabet et al. (2005): 1) prosoma length; 2) prosoma width; 3) length of patella I; 4) length of patella IV; 5) length of tibia I; 6) length of tibia IV; and 7) sex in the case of adults. Once all the data had been recorded, each spider was released in front of its burrow entrance.

### Color polymorphism

After field observations to identify the different morphs of *B. vagans*, 5 adult organisms representative of the varieties in the color patternsobserved were collected. The individuals were taken to the photographic laboratory of El Colegio de la Frontera Sur (Chetumal, Mexico) to be photographed under the same light conditions using a Nikon D7000 (flash Nikon SB-900, lens Nikon 18-105mm, exposure time 1/160, f/9.0, ISO 200, and exposure bias 1.7EV; www.nikon.com).

*B. vagans* presents two variations in color pattern (West 2005): the first (Figure 2A) corresponds to entirely velvet black legs and prosoma, with long orangey red hairs on the abdomen (color pattern called A), and the second variation (Figure 2A') corresponds to the same as the first variation but with an additional wide buff-colored fringe around the prosoma (color pattern called A'). These two patterns of coloration were used in our study, and three new variations of color pattern for *B. vagans* were identified during field work at different villages. One morph presented the same characteristics as pattern A, but also at least a third of the prosoma was colored red-orange (Figure 2B). A second morph presented an overwhelming brown color for the prosoma and legs, while the abdomen presented the typical red color (Figure 2C). A third morph was characterized by a pink or red color for the prosoma, while the abdomen and legs presented a black color with pink or red hairs (Figure 2D).

### Data Analysis

All morphological measurements of adults and juveniles were compared using a Kruskal-Wallis test among the four study sites. All morphometric measurements of adults were compared between the two island populations (both pooled) and the 2 continental populations (both pooled) with a Mann-Whitney *U*-test. All morphometric measurements of juveniles were compared between island populations (both pooled) and continental populations (both pooled) with a Mann-Whitney *U*-test. These analyses were performed with Statistica software version 6.1 (StatSoft Inc., www.statsoft.com). The percentage of individuals belonging to each category of morphs (polymorphism color) was compared with a Maximum Likelihood ratiotest (*G*-test) in each local population.

**Figure 3.**
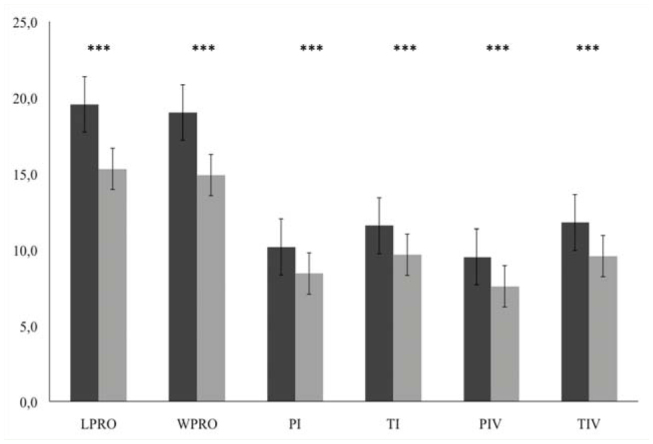
Mean and standard errors (mm) of morphometric measurements (LPRO: length of prosoma; WPRO: width of prosoma; Pl: length of the patella I; Tl: length of tibia; PIV: length of the patella IV; TIV: length of tibia IV) of adult *Brachypelma vagans*. Comparison between island (black bars) and continental (grey bars) populations. Probability associated with Mann-Whitney *U*-test, *** *p* < 0.001. High quality figures are available online.

## Results

### Morphometric analysis

A total of 119 individuals were collected, with a higher number of females (N = 29) and juveniles (N = 88) and just 1 male. Preliminary tests indicated no significant differences (Kruskal-Wallis test) for adults and for juveniles between the two mainland populations (Raudales and Zoh-Lagun ) and between the two island populations (Rancho Guadalupe and El Cedral ) for all characters measured. Consequently, we decided to pool individuals of both mainland populations and individuals of both island populations (adults together and juveniles pooled together in both instances) for further analyses.

**Figure 4.**
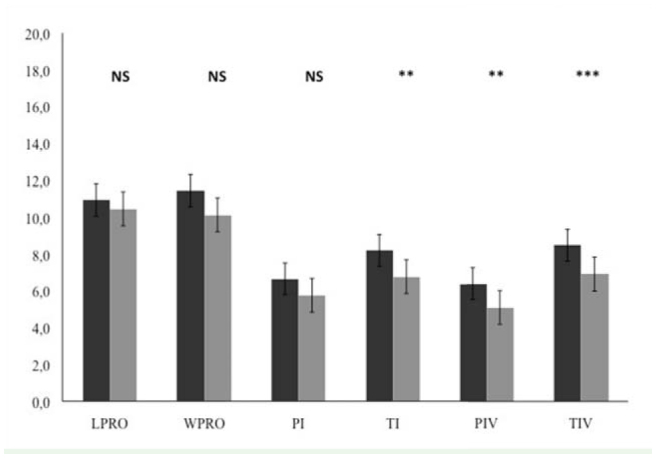
Mean and standard errors (mm) of morphometric measurements (LPRO: length of prosoma; WPRO: width of prosoma; Pl: length of patella I; Tl: length of tibia; PIV: length of patella IV; TIV: length of tibia IV) of juvenile individuals. Comparison between island (black bars) and continental (grey bars) populations. Probability associated with Mann-Whitney *U*-test: NS: not significant, ^**^ *p* < 0.01, ^***^ *p* < 0.001. High quality figures are available online.

All adult individuals on the island were significantly larger than those on the continental locations: length of prosoma: *U*_1,30_ = 16, *p* < 0.001; width of prosoma: *U*_1,30_ = 26, *p* < 0.001; length of patella I: *U*_1,30_ = 17, *p* < 0.001; length of tibia I: *U*_1,30_ = 35, *p* < 0.001; length of patella IV: *U*_1,29_ = 10, *p* < 0.001; and length of tibia IV: *U*_1,29_ = 10, *p* < 0.001 (Figure 3). Analysis of the juveniles showed significant differences, greater for island individuals, between the island and mainland populations only for some leg measurements: length of tibia I: *U*_1,88_ = 615, *p* < 0.01; length of patella IV: *U*_1,88_ = 582, *p* < 0.01; and length of tibia IV: *U*_1,88_ = 551, *p* < 0.001 (Figure 4).

### Color polymorphism

The typical color pattern of *B. vagans* (Figure 2A, A') was the most frequent and significant (Raudales: *G* = 51.3, df = 3, *p* < 0.001; Zoh-Lagun: *G* = 16.6, df = 2, *p* < 0.001; Rancho Guadalupe: *G* = 24.9, df = 1,*p* < 0.001) morph encountered in all populations, but had a great variation in frequency, from 100% in El Cedral to 66% in Zoh-Lagun (Table 1). The individuals in the island populations exclusively presented the typical color pattern (A and A') of *B. vagans* (except for 1 individual in Rancho Guadalupe), whereas in the continental populations the individuals presented all kinds of patterns, as presented in Figure 2 (from 2A to 2D). In the Zoh-Laguna population, up to 34% of individuals were observed with a color pattern different from those previously described for *B. vagans* by West (2005).

**Table 1.**
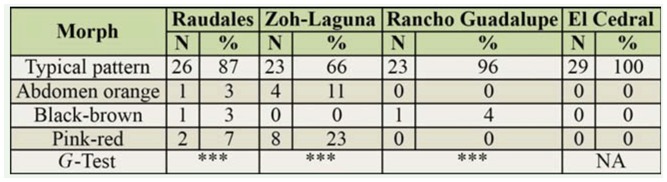
Frequencies of different characteristic morphs (refer to Figure 2) observed for *Brachypelma vagans* in each population. N: number of individuals; %: percentage of individuals; Probability associated with G-test: ^***^ *p* < 0.001, NA: not applied.

## Discussion

Despite the fact that the introduction of *B. vagans* on Cozumel Island is very recent (40 years; Moralez and Cuaron 1999) in relation to evolution time, the results of our study showed clear differences in body size between the mainland and island individuals. Larger bodies were found in island individuals for both juvenile and adult group classes. Other studies have shown that the evolutionary changes for individuals introduced into new habitats (laboratory or natural conditions) can be observed in short time periods (Rice and Hostert 1993; Stockwell et al. 2003; Cox 2004). Fossil records suggest that morphological changes in species that become isolated on islands can occur rapidly. Millien (2006) confirmed that mammals introduced on islands accelerate evolutionary changes over a relatively short time (from some decades to thousands of years). The evolutionary changes range from modifications in body size (e.g., wild population of red deer, *Cervus elaphus*, in Norway), genotypic frequency alterations (e.g., wild populations of *Drosophila* ssp.), and even essential mechanisms such as diapause time or modification of the photoperiod, as in the pitcher plant mosquito ( *Wyeomyia smithii*) (Parmesan 2006). Our study is the first to report significant changes in body size in spiders species introduced into a new isolated habitat, such as an island, over a short period of time. The results showed that, like in other taxonomic groups (mammals, reptiles, insects) (Porter and Geiger 1988; Millien 2006; Kenis et al. 2009), arachnids seem to accelerate their morphological evolution when isolated on an island.

Variation of body size in adaptation to new habitats can result from genetic variations (Birkhof et al. 2006) and environmental adaptations to local conditions, especially temperature (Pinto et al. 2008; Mathys and Lockwood 2011). The short period of time since the introduction of *B. vagans* to Cozumel Island suggests that the differences observed in body size may indicate evolutionary adaptations to the new environmental conditions. Before the introduction of *B. vagans* onto the island, there were no reports of their presence in the area. Its success is probably due to its wide diet and the absence of natural predators. Furthermore, Cozumel Island presents low anthropogenic pressure (low human population with limited movement), which possibly contributed to a better dispersal of juveniles and adults throughout the island, decreasing the possibility of their extermination (Hénaut, personal observation) and illegal trade (Locht et al. 1999). Millien (2006) postulates that, when released on islands, animals such as rodents, lizards, turtles, or insects that are small on the continent tend to become larger than their relatives on the mainland, principally because of lack of predators and reduction of interspecific competition. In contrast, large continentalmammals tend to become smaller on islands due to the availability and access of space and resources (Millien 2006). The increase in body size of *B. vagans* individuals introduced on Cozumel Island most likely resulted from the high availability of food and space in the new habitat and the lack of specific predators, which allowed them to reach sexual maturity later without risk of predation before reproduction (Fox and Czesak 2000). The results of this study are the first to show that individuals of an insular population of tarantula are larger than their mainland relatives.

Another interesting result of our study was the intraspecific color polymorphism reported for the first time in this tarantula species. Several studies refer to the ecological and evolutionary relevance of variation in body morphs in several animal groups, both vertebrate and invertebrate (Oxford and Gillespie 1998, 2001; Oxford 2005; Roulin and Bize 2007; Oxford 2009). It is very common in arthropods, and particularly spiders, to observe both inter- and intra-specific color and pattern variations (Oxford 1998). This has been related to specific environmental conditions in some species groups (Oxford 2009). For instance, in the spiders *Misumena vatia*, *Cytophora citricola*, *Araneus diadematus*, and *Peucetia viradans*, and other species that live in open habitats, a reversible color variation known as crypsis (evolutionary strategy of defense) (Oxford 1998) has been mentioned. Other studies have focused on the relationship between the success of prey capture and color pattern variations in various spider species such as *Micrathena gracilis*, *Nephila pilipes*, and *Gasteracantha cancriformis* (Eberhard 2003; Chou et al. 2005; Gonzaga and Vasconcellos-Nieto 2005). However, the evidence of this function for color variation remains contradictory (Vanderhoff et al. 2008; Gawryszewski and Motta 2012). Other functions of color pattern variations have been studied in crab spiders, *Thomusus onustus*, which use different color morphs as a spectacular strategy of mimicry that works on the visual systems of both predator and prey (Théry and Casas 2002). The variation in color pattern is a very plastic and important characteristic for the development of numerous evolutionary strategies and depends on the biology of each organism. When studying color patterns in predators, it is important to note that the perceived color of an animal depends on the visual system of the organism that observes the light falling on the animal and the substrate where it is placed (Endler 1990). In contrast, different studies have approached color polymorphism by studying the genetic bases (Peckard 1905; Neck 1978; Oxford and Gillespie 1998; Oxford and Gillespie 1996a, b). Research in this field has genetically identified the expression of pigments (ommochromes) as ancestral characters and found pigment in more primitive groups like Araneomophae and Mygalomorphae (Oxford and Gillespie 1998).

Overall, these studies show that the color polymorphism in spider species has a wide range of functions. For *B. vagans*, an active nocturnal predator, the variation in their body colors could serve as a predation strategy to increase hunting success, a camouflage strategy, or simply the emergence of a different phenotype with no definite function. In our study, most of the island's individuals (98%) presented the typical color pattern describe by West (2005). This stability in body color is the result of the founder effect (40 years ago) (Machkour-M'Rabet et al. 2012). In contrast, the mainland populations presented high color polymorphism, with 25% of individuals presenting one of the newly reported color variants. The Zoh-Laguna population presented the highest number of individuals (34%) not having the typical pattern. The most likely explanation for this variation is that Zoh-Laguna is the old and central population of *B. vagans* in this region (Machkour-M'Rabet et al. 2012) and has regular contact with several other local populations from different regions of the south of Mexico. Furthermore, the central position increases the probability of encounters with other individuals, which will thus increase the diversity of color polymorphism. The Raudales population, in contrast, is a geographically isolated population and probably has little contact with other *B. vagans* individuals. This can be explained by the isolation of this locality from the highway and by a permanent network of freshwater lagoons and rivers, offering few possibilities of emigration and immigration for *B. vagans* (Machkour-M'Rabet et al. 2012).

Even though our results concerning the variation of color morphs in *B. vagans* most likely have a biological or ecological explanation, the possibility of a hybridization with close species such as *Brachypelma epicureanum*, distributed in the northern part of Yucatan peninsula, and *B. smithi*, reported in the western part of the Mexican Pacific principally in the state of Guerrero, cannot be excluded (Smith 1994; Platnick 2011). In fact, in captivity it has been observed that different species of *Brachypelma* are able to cross successfully (Hénaut, personal observation). As yet, the genetic situation of these hybrids in captivity is unknown, and no information is available about the possibility of hybridization among *Brachypelma* spp. in the wild.

As mentioned above, the size and color morph variations for a species of tarantula can have different explanations, some more probable than others. Generally, it should be kept in mind that the new population in Cozumel Island probably resulted from the introduction of few individuals generating a founder effect with the resulting genetic consequences, which would increase the impact of any genetic drift. The possible genetic impact of the founder effect on the results cannot be excluded, but the data set would need to be increased and genetic analysis using nuclear markers would need to be performed. The results of our study show *B. vagans* to be an interesting and promising model for evolutionary studies on changes in body size of introduced and isolated individuals and the function of color variation in tarantula species. A more extensive study of island populations of *B. vagans* to understand the invasion scale and the impact of this tarantula species on the local invertebrate fauna is suggested.
